# Combinational Deletions of MGF360-9L and MGF505-7R Attenuated Highly Virulent African Swine Fever Virus and Conferred Protection against Homologous Challenge

**DOI:** 10.1128/jvi.00329-22

**Published:** 2022-07-06

**Authors:** Mingyang Ding, Wen Dang, Huanan Liu, Fan Xu, Huaguo Huang, Yongjie Sunkang, Tao Li, Jingjing Pei, Xiangtao Liu, Yong Zhang, Haixue Zheng

**Affiliations:** a College of Veterinary Medicine, Gansu Agricultural University, Lanzhou, China; b State Key Laboratory of Veterinary Etiological Biology, Lanzhou Veterinary Research Institute, Chinese Academy of Agricultural Sciences, Lanzhou, China; c National Foot and Mouth Diseases Reference Laboratory, Lanzhou Veterinary Research Institute, Chinese Academy of Agricultural Sciences, Lanzhou, China; d Key Laboratory of Animal Virology of Ministry of Agriculture, Lanzhou Veterinary Research Institute, Chinese Academy of Agricultural Sciences, Lanzhou, China; e College of Veterinary Medicine, Northwest A&F University, Yangling, Shaanxi, China; University of Illinois at Urbana Champaign

**Keywords:** ASFV, MGF360-9L, MGF505-7R, attenuated phenotype, immune response, protective efficacy

## Abstract

Multigene family (MGF) gene products are increasingly reported to be implicated in African swine fever virus (ASFV) virulence and attenuation of host defenses, among which the MGF360-9L and MGF505-7R gene products are characterized by convergent but distinct mechanisms of immune evasion. Herein, a recombinant ASFV mutant, ASFV-Δ9L/Δ7R, bearing combinational deletions of MGF360-9L and MGF505-7R, was constructed from the highly virulent ASFV strain CN/GS/2018 of genotype II that is currently circulating in China. Pigs inoculated intramuscularly with 10^4^ 50% hemadsorption doses (HAD_50_) of the mutant remained clinically healthy without any serious side effects. Importantly, in a virulence challenge, all four within-pen contact pigs demonstrated clinical signs and pathological findings consistent with ASF. In contrast, vaccinated pigs (5/6) were protected and clinical indicators tended to be normal, accompanied by extensive tissue repairs. Similar to most viral infections, innate immunity and both humoral and cellular immune responses appeared to be vital for protection. Notably, transcriptome sequencing (RNA-seq) and quantitative PCR (qPCR) analysis revealed a regulatory function of the mutant in dramatic and sustained expression of type I/III interferons and inflammatory and innate immune genes *in vitro*. Furthermore, infection with the mutant elicited an early and robust p30-specific IgG response, which coincided and was strongly correlated with the protective efficacy. Analysis of the cellular response revealed a strong ASFV-specific interferon gamma (IFN-γ) response and immunostaining of CD4^+^ T cells coupled with a high level of CD163^+^ macrophage infiltration in spleens of vaccinated pigs. Our study identifies a new mechanism of immunological regulation by ASFV MGFs that rationalizes the design of live attenuated vaccine for implementation of improved control strategies to eradicate ASFV.

**IMPORTANCE** Currently, the deficiency in commercially available vaccines or therapeutic options against African swine fever constitutes a matter of major concern in the swine industry globally. Here, we report the design and construction of a recombinant ASFV mutant harboring combinational deletions of interferon inhibitors MGF360-9L and MGF505-7R based on a genotype II ASFV CN/GS/2018 strain currently circulating in China. The mutant was completely attenuated when inoculated at a high dose of 10^4^ HAD_50_. In the virulence challenge with homologous virus, sterile immunity was achieved, demonstrating the mutant’s potential as a promising vaccine candidate. This sufficiency of effectiveness supports the claim that this live attenuated virus may be a viable vaccine option with which to fight ASF.

## INTRODUCTION

African swine fever virus (ASFV) is the causative agent of African swine fever (ASF), a contagious hemorrhagic disease of wild and domestic pigs. The disease manifestations differ, ranging from chronic or subclinical forms to acute hemorrhagic forms that can result in sudden death of the infected hosts in few days (1). Since its identification in Kenya in 1921, ASFV has remained endemic in Africa. Later, ASFV genotype I circulated transiently and regionally in Europe, Russia, the Caribbean, and South America but was quickly eradicated in those areas by the mid-1990s, with the exception of the island of Sardinia, where it has remained, circulating endemically (2). In 2007, ASFV genotype II was reintroduced into the Republic of Georgia and continued to spread through the Caucasus region and subsequently into the Russian Federation and Eastern Europe (3). More importantly, in August 2018, Georgia-07-like genotype II ASFV emerged for the first time in the People’s Republic of China and soon spread to neighboring countries across Asia, posing a huge threat to the pig industry globally (4). In 2021, two nonhemadsorbing genotype I isolates were isolated from domestic pig farms in two provinces in China. Both isolates were phylogenetically related to NH/P68 and OURT88/3, two genotype I ASFVs isolated in Portugal in the last century, and were characterized by low virulence and efficient transmissibility in pigs, presenting more problems and challenges for the control and prevention of ASF in China (5).

ASFV is characterized by high complexity, evidenced by its long double-stranded DNA (dsDNA) genome of 170 to 190 kb, encoding more than 150 proteins with diverse and mysterious functions in various stages of ASFV’s life cycle. Recent cryo-electron microscopy (cryo-EM) structural analysis of ASFV revealed the most interesting feature, its unique multiple-layer icosahedral structure. In detail, the ASFV capsid structure is built from 17,280 proteins, including one major (p72) and four minor (M1249L, p17, p49, and H240R) capsid proteins organized into pentasymmetrons and trisymmetrons (6–8). Moreover, some work has highlighted viral interactions with the host nucleus, providing new antiviral targets against ASFV replication (9, 10). Those findings advance the fight against the disease.

Interestingly, the functions of many ASFV genes are still elusive, especially those located in the left and right variable regions (LVR and RVR), referred to as multigene families (MGFs) of paralogous genes. Five MGFs (MGF100, MGF110, MGF300, MGF360, and MGF505, named based on the average amino acid length of the proteins they encode) have been characterized, and they displayed genetic diversity with relatively high mutation rates and complexity in gene contents (11). The implications of MGFs for ASFV virulence, immunogenicity, and more importantly, immune evasion have been increasingly documented. ASFV MGF360 and MGF505 (MGF360/505) gene products may either directly or indirectly suppress a type I interferon (IFN) response, as analyzed by using a swine cDNA microarray (12). A high-throughput functional screening identified ASFV protein A276R (MGF360-15R) as an antagonist of IFN-β production via both the Toll-like receptor 3 (TLR3) and cytosolic pathways (13). Moreover, ASFV MGF505-7R (A528R) could inhibit the induction of both NF-κB- and interferon-regulated factor 3 (IRF-3)-mediated type I IFN responses (13). Very recently, it was discovered that MGF505-7R could negatively regulate the cGAS-STING-mediated type I IFN response by either degrading STING or reducing STING expression (14). In parallel, MGF505-7R was found to downregulate interleukin-1β (IL-1β) and type I IFN production by interacting with IκB kinase α (IKKα) in the IKK complex and binding to NLRP3, as well as inhibiting the nuclear translocation of IRF3 (15). In both studies, a recombinant MGF505-7R deletion mutant displayed an attenuated phenotype in pigs. Moreover, MGF360-9L was reported to antagonize the interferon beta (IFN-β) signaling pathway by degrading the key components of the JAK-STAT pathway. Deletion of MGF360-9L conferred attenuated virulence on ASFV and boosted the type I interferon response *in vitro* and *in vivo* (16). Those findings consistently relate MGFs to viral pathogenesis and immune evasion, further highlighting the importance of deciphering the role of MGFs in ASFV’s biology.

There is no safe and efficacious vaccine available for control of ASF so far. Previous studies have shown that protective immunity against ASF is achievable when pigs are immunized with live attenuated viruses that are naturally isolated or genetically manipulated (17–20), prompting us to develop a highly effective ASFV vaccine by combinational deletions of two robust ASFV interferon inhibitors, MGF360-9L and MGF505-7R. The results of our investigation of the pathogenesis and protective efficacy of the ASFV-Δ9L/Δ7R mutant revealed that the mutant lost virulence, being unable to cause severe clinical signs in pigs, but elicited an effective protective response against a lethal challenge with the parental ASFV. Extensive studies demonstrated that the two deletions in the mutant not only synergized in boosting a more pronounced interferon response and higher expression of inflammatory and innate immune genes *in vitro* but also elicited an ASFV-specific IFN-γ response together with a p30-specific IgG response, which coincided with protective efficacy. Together with known roles of MGFs, our study marks a potential live attenuated ASFV vaccine targeting innate and both humoral and cellular immune responses that can contribute to ASF preventive strategies.

## RESULTS

### Conservation of MGF360-9L and MGF505-7R genes across different ASFV isolates.

**FIG 1:**
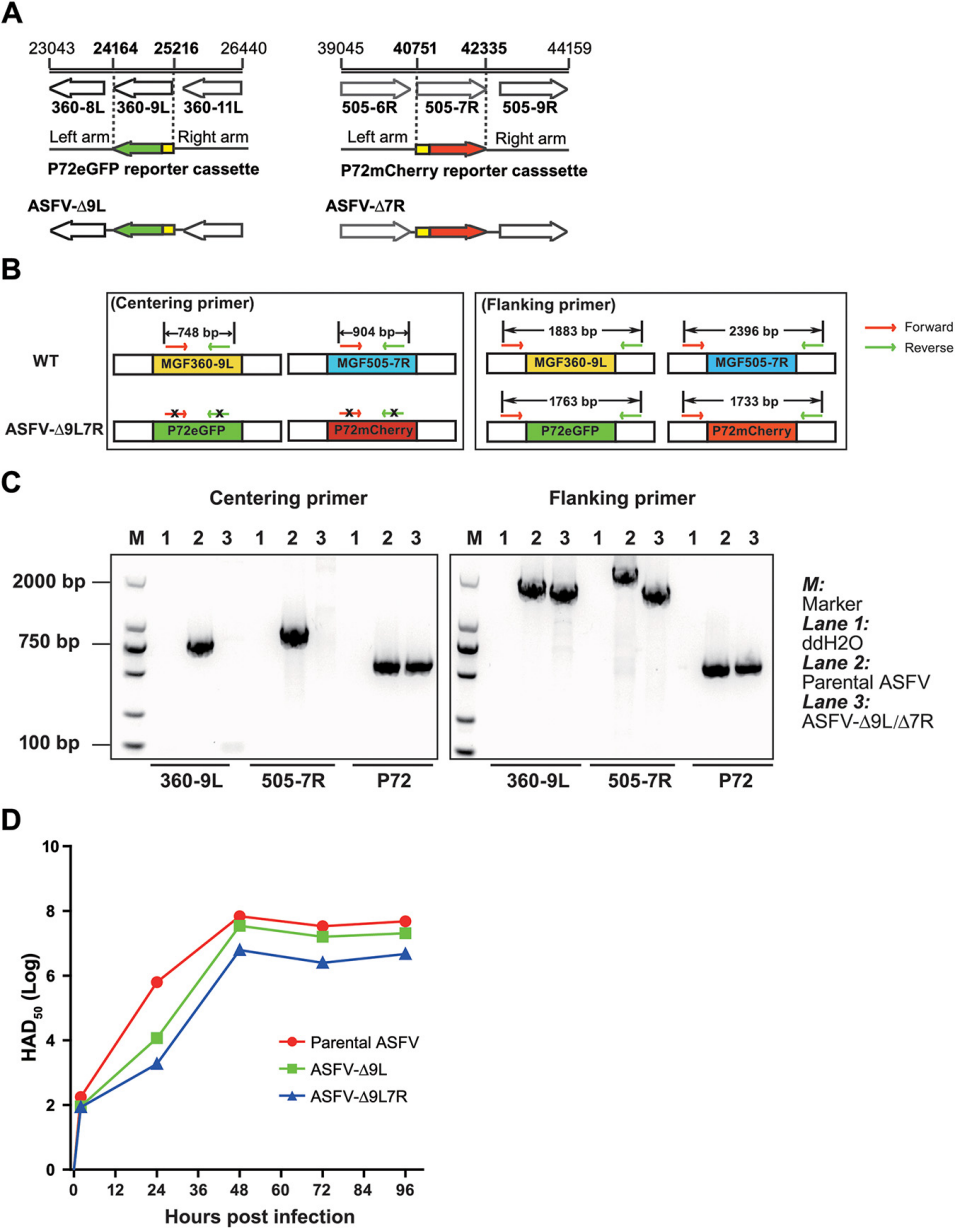
Construction and characterization of a double-gene-deletion ASFV-Δ9L/Δ7R mutant. (A) Schematic diagrams of recombinant transfer vectors pASFV-Δ9L and pASFV-Δ7R. Open reading frames are indicated by arrows, with nucleotide positions labeled above. The deleted MGF360-9L and MGF505-7R were replaced with the p72eGFP and p72mCherry reporter gene cassettes, respectively. (B) Schematic diagrams of designs for centering primers and flanking primers located within or flanking the target gene, respectively. The expected DNA sizes of PCR products are indicated above. The X symbols in the left panel indicate that no bands were obtained by PCR. WT, wild type. (C) PCR analysis of ASFV-Δ9L/Δ7R using two sets of primer pairs, with the p72 primer pair used as an indicator of loading ASFV genomic DNA. ddH_2_O, double-distilled water. (D) Replication kinetics of parental ASFV, ASFV-Δ9L mutant, and ASFV-Δ9L/Δ7R mutant. BMDM were infected with ASFV at an MOI of 0.5. Following the indicated durations (2 h, 24 h, 48 h, and 96 h), whole-cell cultures were subjected to repeated freeze-thawing processes. Virus titers in the supernatant were titrated by HAD_50_.

The open reading frames (ORFs) of MGF360-9L and MGF505-7R are in the LVR of ASFV strain CN/GS/2018, with positions on the forward strand from 24164 to 25216 and 40751 to 42335, respectively (Fig. 1A). Aiming to decipher the degrees of conservation of those two genes, a subset of all sequenced isolates of ASFV representing mainly genotypes I, II, and IV from domestic pig, wild pig, and tick sources were included in the present analysis (21). MGF360-9L is highly conserved across genotype I and II isolates, albeit there is an insertion of 17 amino acids at the N terminus for genotype II virus isolates from Estonia, Russia, and Poland and several amino acid substitutions near the C terminus of genotype I isolates. It should be noted that MGF360-9L of genotype IV and other isolates demonstrate a different pattern of amino acid sequences than genotype I and II isolates (Fig. S1 in the supplemental material). Surprisingly, genotype I, II, and IV isolates display different patterns of the amino acid sequences of MGF505-7R, with less sequence identity (Fig. S2).

### Construction and characterization of the recombinant ASFV-Δ9L/Δ7R mutant.

The overall strategy to design the ASFV-Δ9L/Δ7R mutant is shown in Fig. 1A. Of note, a single-gene-deletion ASFV-Δ9L mutant was preferentially constructed from the highly pathogenic ASFV CN/GS/2018 isolate by replacing the MGF360-9L gene with a reporter gene cassette containing the enhanced green fluorescent protein (eGFP) gene under the ASFV p72 late gene promoter (p72eGFP gene cassette). Subsequently, ASFV-Δ9L was applied in a second homologous recombination process to produce a double-gene-deletion ASFV-Δ9L/Δ7R mutant with MGF505-7R replaced by a p72mCherry-encoding reporter gene cassette. The characterization of the genetic modification of the ASFV-Δ9L/Δ7R mutant was performed by PCR analysis (Fig. 1B). Using centering primer pairs targeting MGF360-9L and MGF505-7R, PCR analysis detected no bands in the ASFV-Δ9L/Δ7R mutant, indicating the combinational deletions of the two target genes (Fig. 1C, left). PCR analysis to further evaluate the accuracy of the genetic modifications detected differential band sizes using flanking primer pairs in the parental ASFV and the ASFV-Δ9L/Δ7R mutant, suggestive of insertions of reporter gene cassettes in the ASFV-Δ9L/Δ7R mutant (Fig. 1C, right). Moreover, the PCR fragments were cloned and sequenced. Except for the designed genetic modifications, no genetic changes were detected in the target regions (Fig. S3 and S4).

Next-generation sequencing (NGS) analysis of the mutant genome sequence relative to the parental ASFV genome sequence to evaluate the integrity of the genome of the mutant displayed the following differences between these two viruses: (i) eight nucleotide deletions, all in noncoding segments of the genome; (ii) one nucleotide insertion of 4 bp of GGGG at position 20428 in a noncoding segment; (iii) a nucleotide mutation of T to C at position 34365 in a noncoding segment; (iv) five nucleotide mutations at positions 75867 (ORF M1249L), 116058 (ORF G1211R), 123138 (ORF CP2475L), 138410 (ORF D205R), and 141832 (ORF D1133L) resulting in residue substitutions; and (v) a nucleotide mutation of A to G at position 117734 resulting in a silent mutation in ORF CP2475L (Table 1). Collectively, the mutant did not accumulate any significant mutations during the process of homologous recombination and the consequent limiting dilution steps.

**TABLE 1:** Summary of differences between the full-length genome sequence of ASFV-9L/7R and the parental ASFV CN/GS/2018

NPN^*a*^	Region or ORF, description of modification^*b*^
27	NCR, deletion of C
1565	NCR, deletion of A
6821	NCR, deletion of A
16475	NCR, deletion of G
18629	NCR, deletion of G
20428	NCR, insertion of GGGG
21027	NCR, deletion of C
22622	NCR, deletion of A
34365	NCR, T to C
54119	NCR, deletion of T in NCR
75867	M1249L, T to C (Asp1062Gly)
116058	G1211R, A to G (Thr1093Ala)
117734	CP2475L, A to G (Gly2190Gly), SM
123138	CP2475L, A to G (Ile389Thr)
137410	D205R, A to C (Asn102Thr)
141832	D1133L, T to C (His510Arg)

a NPN, nucleotide position number based on the sequence of parental strain ASFV CN/GS/2018.

b NCR, noncoding region; SM, the nucleotide modification caused a silent mutation.

### The ASFV-Δ9L/Δ7R mutant showed reduced replication *in vitro*.

The *in vitro* growth characteristics demonstrated that the ASFV-Δ9L/Δ7R mutant showed relatively slower kinetics of replication, reaching a plateau at 48 h postinfection (hpi) with a lower peak virus titer than the ASFV-Δ9L and parental ASFV strains (Fig. 1D). Collectively, the ASFV-Δ9L/Δ7R mutant exhibited attenuated replication fidelity in bone marrow-derived macrophages (BMDM) in relation to that of the parental ASFV.

### Inoculation with the mutant displayed reduced virulence but provided protection against lethal homologous challenge.

**FIG 2:**
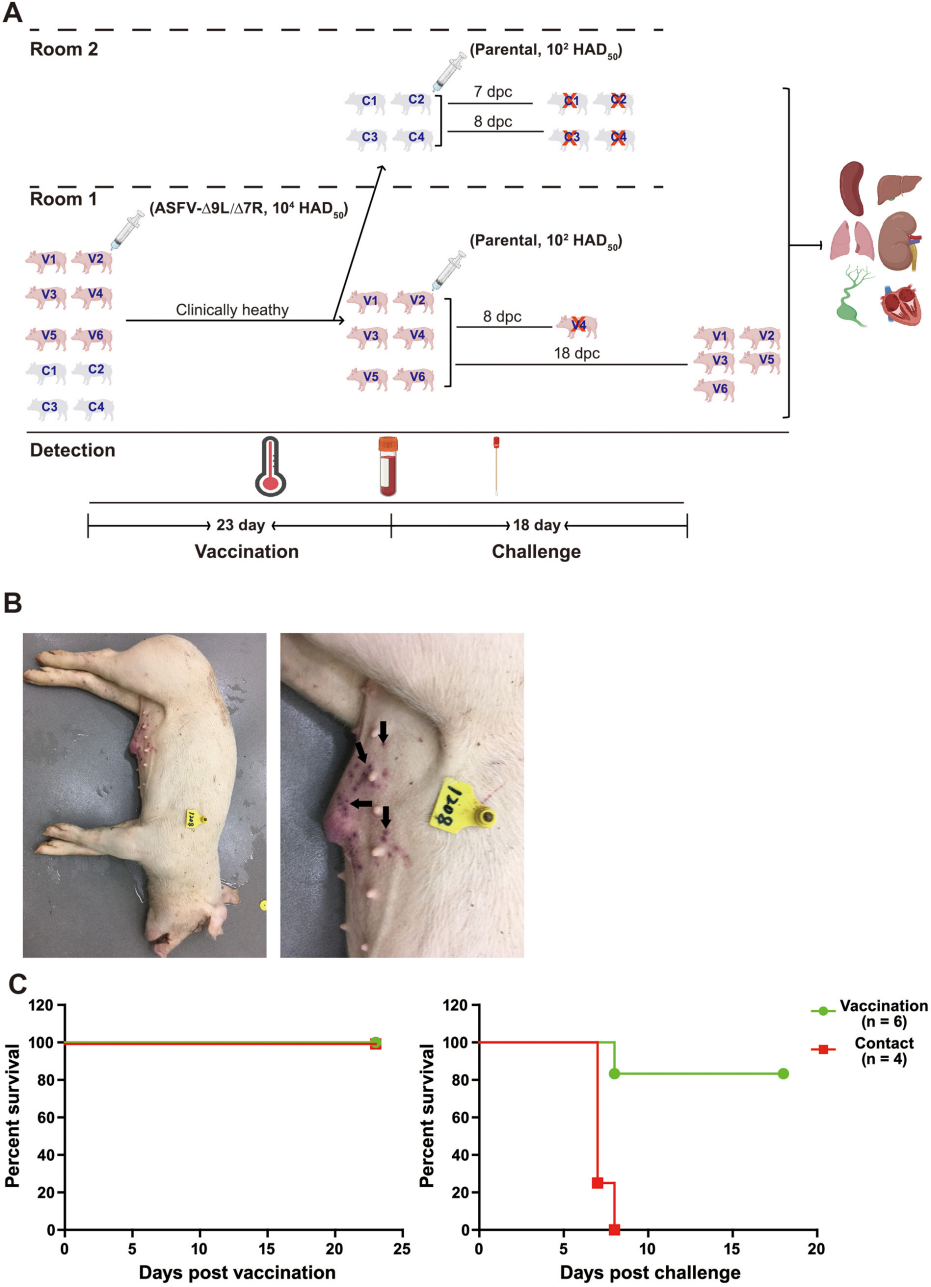
Assessment of virulence of ASFV-Δ9L/Δ7R and its protective efficacy against parental ASFV challenge. (A) Schematic diagram of design and outcomes of animal experiment. Four naive pigs (C1, C2, C3, and C4) were placed in direct contact with six pigs (V1, V2, V3, V4, V5, and V6) intramuscularly inoculated with 10^4^ HAD_50_ of ASFV-Δ9L/Δ7R mutant in room 1. At 23 days postvaccination, the four contact pigs were transferred to a separate room (room 2) for a further challenge experiment. All the pigs were challenged with 10^2^ HAD_50_ of highly virulent parental ASFV CN/GS/2018 and monitored for an extra 18 days. The four contact pigs either died or were euthanized *in extremis* between 7 and 8 days postchallenge (dpc). In contrast, only one vaccinated pig (V4) died, at 8 dpc. Rectal temperature was monitored daily. EDTA plasma samples, serum samples, and swab samples were collected at 2-day intervals. At necropsy, the indicated tissues were acquired. (B) Observational clinical signs of ASVF in contact animal C3. Arrows indicate representative cyanosis and necrotic lesions on the skin of the abdomen. (C) Descriptive survival outcomes of the pigs after vaccination and challenge.

To evaluate the degree of attenuation provoked by MGF deletions and the possible transmission of the ASFV-Δ9L/Δ7R mutant via direct contact, four naive pigs (contact pigs; C1, C2, C3, and C4) were housed together with six pigs (vaccinated pigs; V1, V2, V3, V4, V5, and V6) experimentally inoculated with 10^4^ 50% hemadsorption doses (HAD_50_) of ASFV-Δ9L/Δ7R (Fig. 2A). Surprisingly, all 6 vaccinated animals presented no obvious ASFV-related clinical signs during a 23-day observational period, preliminarily indicating the attenuated phenotype *in vivo*. In the subsequent challenge experiment, the four contact pigs and six vaccinated pigs were placed in separate rooms so that the possibility of environmental cross-contamination would be limited. After parental ASFV challenge, the four contact animals developed fever and ASFV-related clinical signs, namely, diarrhea, anorexia, skin redness, and cyanotic areas mainly in distal limbs and abdomen, as early as 3 days postchallenge (dpc). The clinical signs of the disease worsened progressively over time, and the animals either died or were euthanized *in extremis* by 7 or 8 dpc (Fig. 2B). Conversely, five of the six vaccinated pigs were protected after parental ASFV challenge, with the exception being animal V4. It presented with several clinical appearances and was euthanized with no hope of recovery at 8 dpc (Fig. 2C).

**FIG 3:**
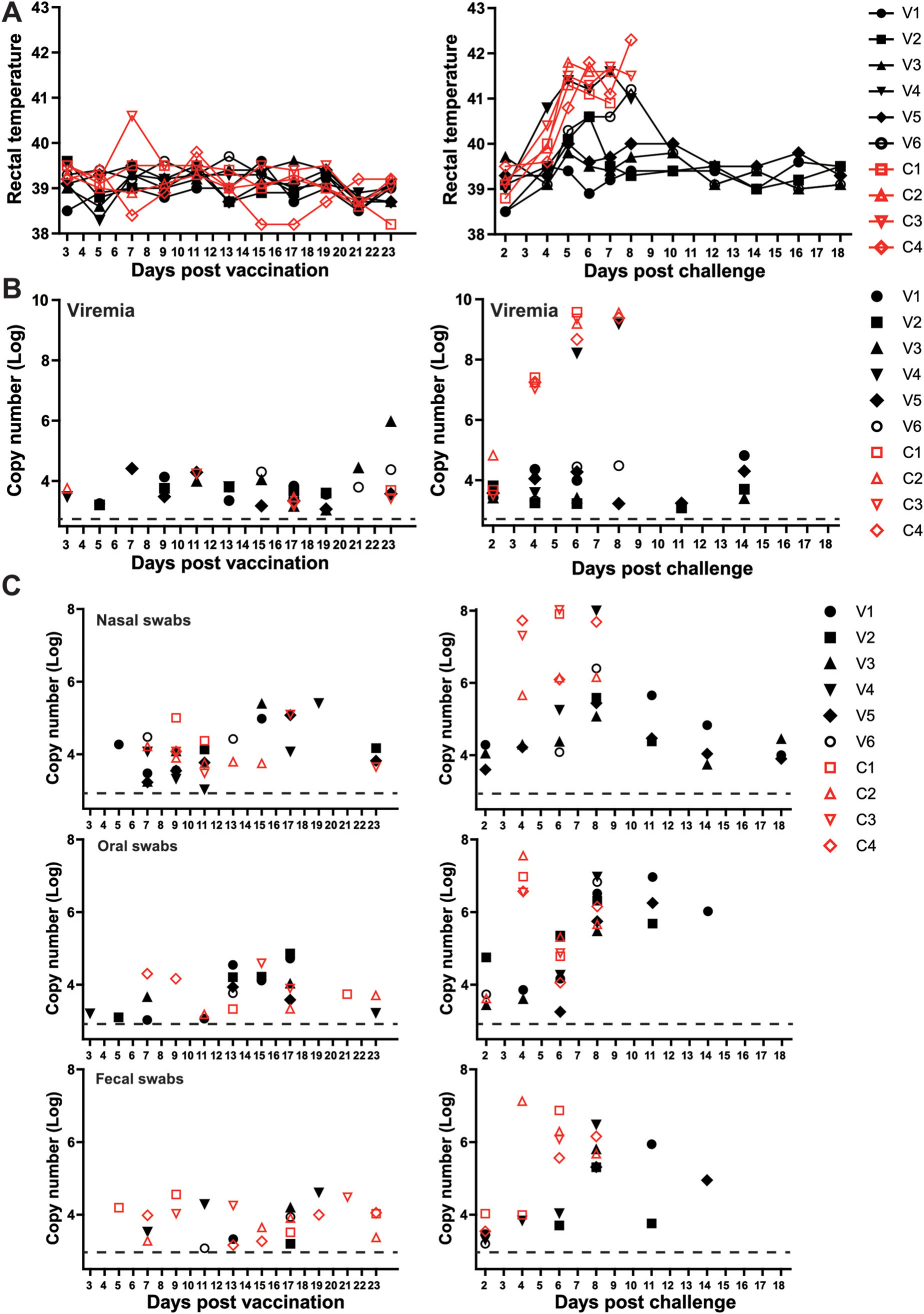
Rectal temperatures, viremia titers, and virus shedding. (A) Rectal temperatures of pigs vaccinated with 10^4^ HAD_50_ of ASFV-Δ9L/Δ7R (*n* = 6, black) or exposed through direct contact (*n* = 4, red) (left) and subsequently challenged with 10^2^ HAD_50_ of highly virulent parental ASFV (right). Rectal temperature of ≥40°C was defined as fever. (B) Virus titers in EDTA plasma samples. Values are expressed as log_10_ copy numbers per milliliter. (C) Virus shedding. Swabs were soaked in phosphate-buffered saline (PBS) medium overnight. The following day, the fluids were vortexed, repeatedly freeze-thawed, clarified, and subjected to copy number detection.

The results from the vaccination experiment demonstrated that vaccinated animals remained clinically healthy, with stable body temperature, remarkably low viremia titers, and almost undetectable virus shedding (Fig. 3A, B, and C, left, black lines and symbols). Similarly, contact pigs demonstrated normal temperature and feedings, (Fig. 3A, B, and C, left, red lines and symbols), implying a low possibility of direct contact transmission. Of note, moderately high levels of ASFV genome copies were occasionally detected in the swab samples from contact pigs (Fig. 3C, left). This is partially because of the natural explorative behavior of domestic pigs, resulting in a high chance for ASFV to transmit from pigs to pigs in secretions. Importantly, in the challenge experiment, four of the six vaccinated pigs remained clinically healthy, accompanied by low levels of ASFV DNA in blood and virus shedding in swabs. Acute fever in animal V6 was transient (5 to 8 dpc) and subsided without any complications. Animal V4 displayed fever starting from as early as 3 dpc, and clinical signs of anorexia, depression, and vomiting were observed prior to euthanasia at 8 dpc. Simultaneously, as anticipated, contact animals had a fever starting from 4 dpc and developed high levels of viremia, with substantially increased viral shedding prior to or at euthanasia at 7 and 8 dpc for reasons of animal welfare (Fig. 3A, B, and C, right, red symbols and lines).

### The ASFV-Δ9L/Δ7R inoculation protected pigs from lesions as observed by postmortem and histological analysis.

**FIG 4:**
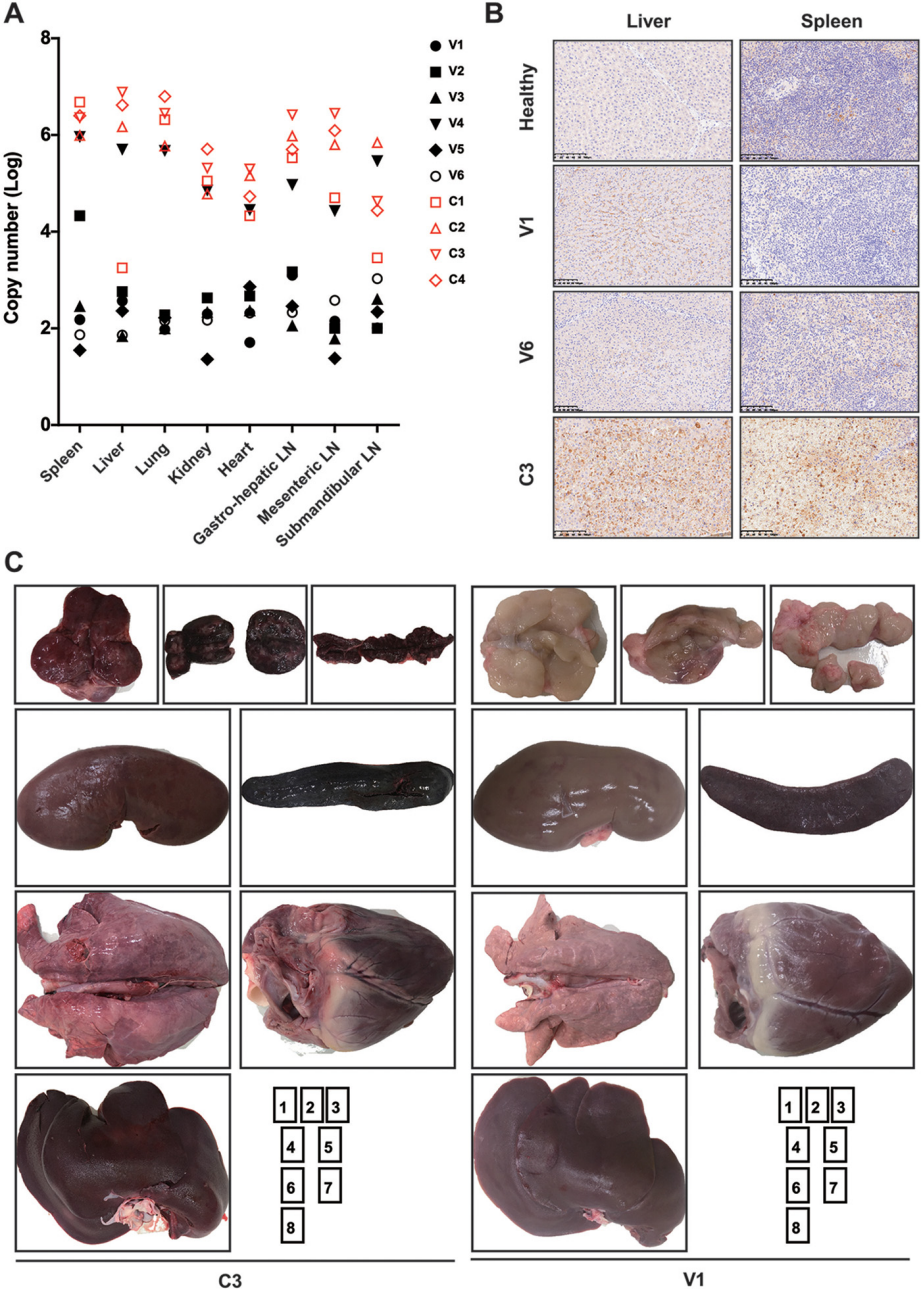
Correlation between viral loads and postmortem lesions. (A) Virus titers in tissues from vaccinated pigs (black, *n* = 6) and contact pigs (red, *n* = 4). Totals of 30 mg of indicated types of tissue samples were homogenized, vortexed, clarified, and subjected to copy number detection. (B) Immunostaining of ASFV p72 antigens in the livers and spleens of one healthy animal, two vaccinated animals (V1 and V6), and one contact animal (C3). Nuclear staining of cells in the contact pig is almost completely absent due to advanced autolysis. (C) Comparative postmortem lesions. The images show representative organs from contact pig C3 (left) and vaccinated pig V1 (right) as follows: 1, submandibular lymph node; 2, gastrohepatic lymph node; 3, mesenteric lymph node; 4, kidney; 5, spleen; 6, lung; 7, heart; and 8, liver.

ASFV infects cells of the mononuclear-phagocytic system, including differentiated fixed tissue macrophages and specific lineages of reticular cells. Thus, ASFV infection is associated with extensive damage in affected tissues (22–24). In analyses performed to decipher the correlation between viral loads and tissue damage, all the vaccinated pigs except animal V4 displayed low ASFV DNA loads, of approximately 10^2^ copies, in the indicated tissues, as anticipated. In contrast, ASFV replicated to much higher levels in contact animals, with increases of 2- to 4-log copies (Fig. 4A). Consistently, the p72 major capsid protein of ASFV was detected in the tissue samples of liver and spleen examined. Strong immunostaining, together with advanced autolysis, was observed in liver samples of contact animal C3 (Fig. 4B, left). The characterization of infected cell types was unattainable, due to severe autolysis. Mild immunostaining was observed intracellularly and extracellularly in liver samples of vaccinated animals V1 and V6, with cells resembling macrophages and limited numbers of hepatocytes being immunolabelled. The immunostaining patterns of spleen samples paralleled the results for liver samples, despite the fact that extremely mild diffuse background staining was observed in the spleen of the healthy animal examined, while such unspecific staining was not observed in the liver of the healthy animal examined (Fig. 4B, Healthy).

At necropsy, all four contact pigs had developed comparable gross lesions at the terminal stages of disease. Our postmortem findings showed that lymph nodes from contact animal C3 were consistently hemorrhagic and enlarged (Fig. 4C, left), whereas in the vaccinated pigs, they were white/pink in color without inflammation (Fig. 4C, right; Fig. S3). Renal lesions varied from mild cortical petechia to renomegaly with diffuse hemorrhage and congestion, while the kidneys in vaccinated pigs were uniformly colored and textured (Fig. 4C; Fig. S3). As ASFV replication occurred at low levels in tissue samples from vaccinated animals, the degrees of tissue involvement and resulting tissue damage were much less severe.

**FIG 5:**
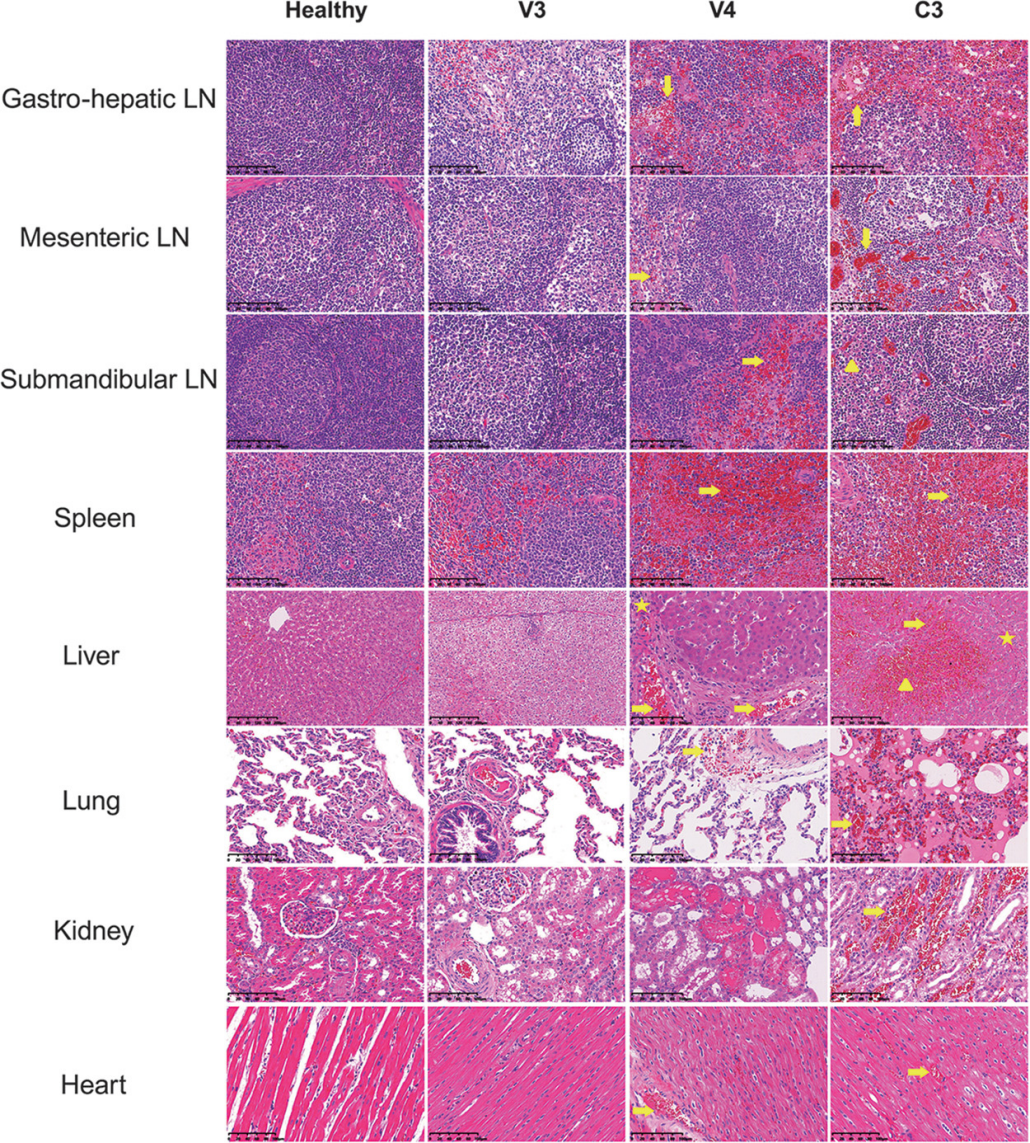
Characterization of histopathological lesions. Representative histopathological lesions in different tissue samples of a healthy pig (far left), vaccinated pigs V3 (survived) and V4 (died), and contact pig C3. Arrows indicate severe acute and diffuse hemorrhages and large numbers of karyorrhectic cells. Triangles indicate lymphoid depletion and loss of lymphocytes. Stars indicate infiltration of inflammatory cells.

Histological examination revealed mild to advanced autolysis in the tissue samples of contact pig C3, as well as the vaccinated but not protected animal V4. The tissue morphology remained intact, but poor nuclear staining was observed, especially in the context of the liver, maybe due to severe karyorrhexis. More detailed observation revealed that severe hyperemia and multifocal diffuse hemorrhage were apparent in all tissue samples except those from the heart. Diffuse lymphoid depletion and loss of lymphocytes were most present in the spleen and lymph nodes. Multifocal infiltration of multiple cell types, mainly neutrophils and monocytes, was predominantly seen in the liver. In contrast, in the vaccinated and protected animal V3, ASFV-related tissue damage was significantly less severe (Fig. 5). Based on the severity, these histological changes were scored from 0 to 5. The histological scores of contact group animals (C3 and C4) were mostly 4 and 5, with a score of 2 or 3 for heart tissue samples and submandibular lymph node tissue samples. In contrast, tissue samples of vaccination group animals (V1, V2, V3, and V5) were mainly scored from 0 to 3, relatively lower than the scores for the same types of tissue samples from the contact group (Fig. 5).

### ASFV p30-specific IgG responses correlate with protective efficacy.

**FIG 6:**
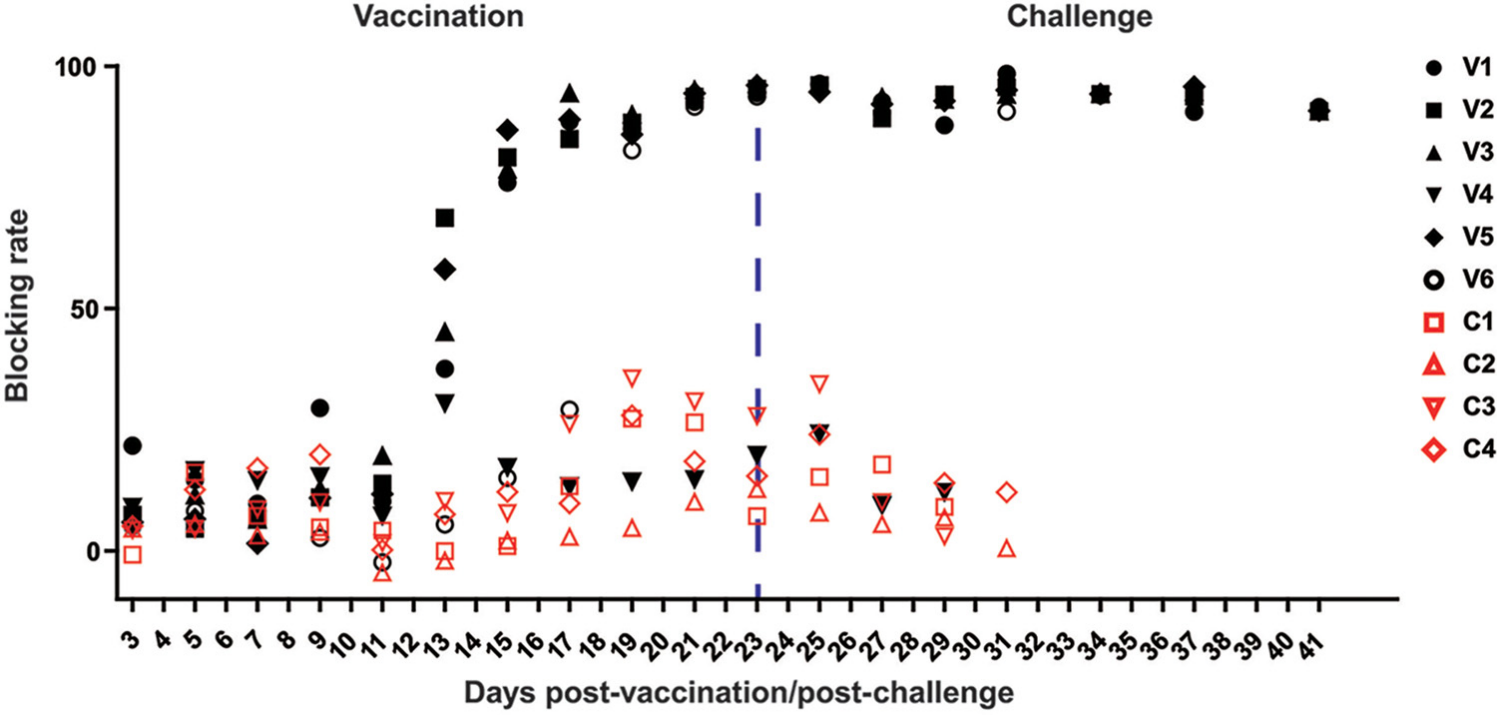
Longitudinal p30 IgG responses in vaccinated (black) versus contact (red) pigs.

Monitoring ASFV p30-specific IgG production kinetics revealed that p30-specific antibodies in four of six vaccinated pigs started to climb at day 13 postvaccination, reached peak titers within 4 days, and remained stable (Fig. 6, black). Animal V6 showed relatively delayed production kinetics but reached the same level of peak titers afterwards. Interestingly, in the case of animal V4, the antibody levels were low and did not differ from those in the contact group at any of the time points evaluated (Fig. 6, red). Passive transfer of ASFV antibodies alone was sufficient to protect pigs from lethal ASFV infection, highlighting the role of humoral immunity in viral clearance (25–27). It was noticeable that the p30-specific IgG responses correlated with protective efficacy after ASFV challenge, as animal V4 was the only one among the six vaccinated pigs that failed to develop an ASFV p30-specific antibody response.

### The ASFV-Δ9L/Δ7R mutant can induce a more pronounced innate immune response *in vitro*.

**FIG 7:**
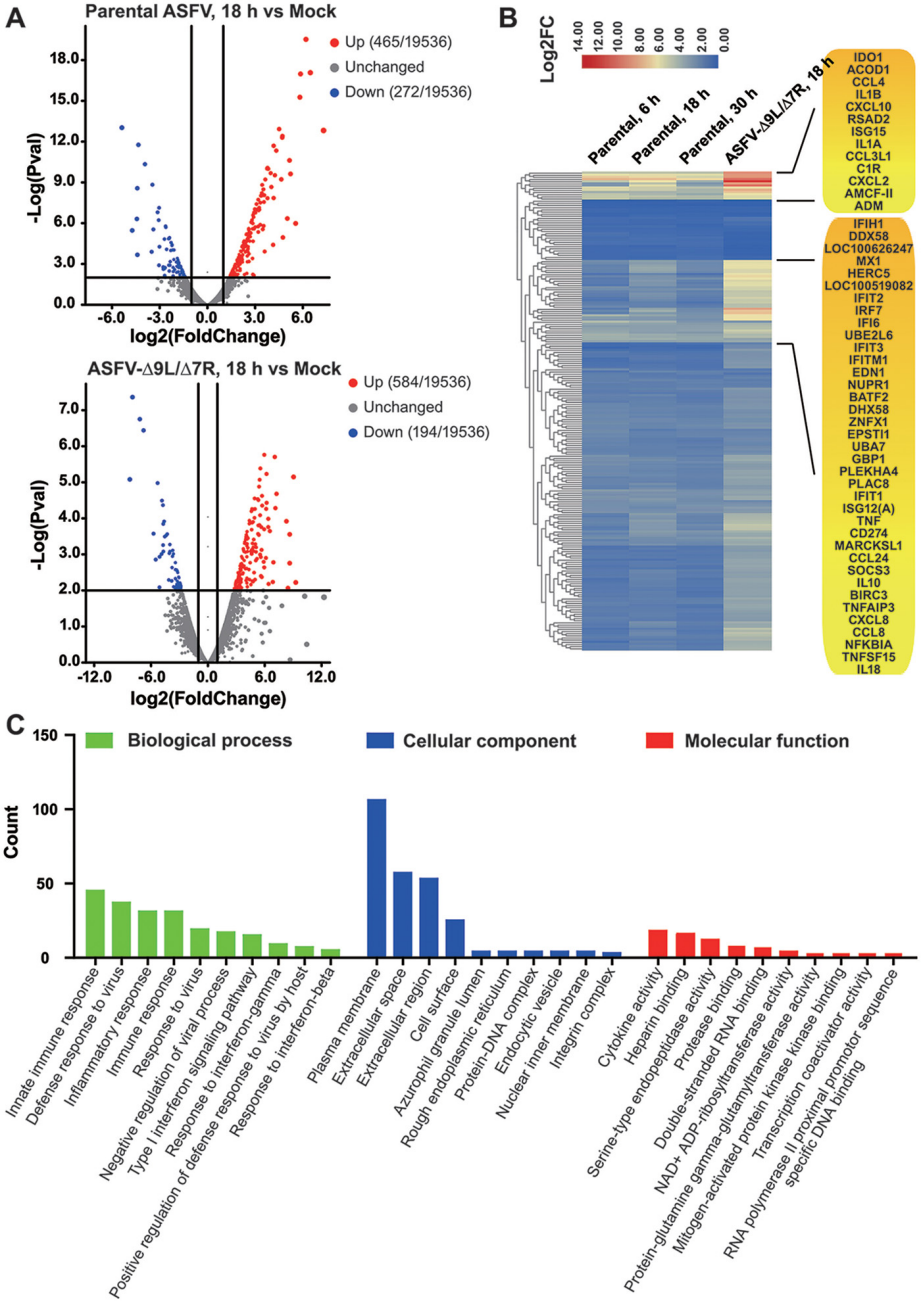
ASFV-Δ9L/Δ7R induced a more pronounced innate immune response *in vitro*. BMDM were mocked infected or infected with parental ASFV and ASFV-Δ9L/Δ7R at an MOI of 0.5. Following the indicated durations (6 h, 18 h, and 30 h), cell cultures were subjected to RNA-seq analysis. (A) Volcano plot of gene changes in ASFV-infected BMDM compared to the expression in mock-infected BMDM. Red dots and blue dots denote upregulated and downregulated DEGs (*P* < 0.01, log_2_(fold change) > 1 or < −1), respectively. (B) Hierarchical clustering of the DEGs identified in ASFV-infected BMDM. A total of 221 genes implicated in the innate immune response were probed over time and are displayed in a heat map. Each panel represents a particular gene, and the color depicts the fold change (FC) at the indicated time points. (C) Histogram of significantly enriched GO classifications of upregulated DEGs. The top 10 upregulated GOTerms involved in the three main categories, namely, biological process, molecular function, and cellular component, are ranked based on the counts of upregulated DEGs in ASFV-Δ9L/Δ7R-infected BMDM compared to parental ASFV-infected samples at 18 h. The *y* axis indicates the number of DEGs in a specific category.

Previous studies have identified MGF360-9L and MGF505-7R as antagonists of the innate immune response, with functions involving immune evasion and virulence, as deletion of the respective gene boosted the innate immune response *in vitro* and *in vivo*. Herein, the issue of whether the combinational deletions of MGF360-9L and MGF505-7R could exert a synergistic effect in boosting the innate immune response was well addressed. The transcriptome analysis demonstrated that ASFV-Δ9L/Δ7R infection induced more differentially expressed genes (DEGs), especially upregulated DEGs with comparably higher overall expression levels, than parental ASFV infection did. A total of 737 DEGs were identified in mock- and parental-ASFV-infected BMDM, among which 465 were upregulated and 272 were downregulated (Fig. 7A, top). In contrast, 778 DEGs were identified in mock- and ASFV-Δ9L/Δ7R-infected BMDM, with 584 DEGs being upregulated and 194 DEGs being downregulated (Fig. 7A, bottom). Hierarchical clustering of DEGs of the innate immune response demonstrated that ASFV-Δ9L/Δ7R infection resulted in higher transcriptional induction of the interferon response, especially IFN-induced genes, such as IFN-induced proteins with tetratricopeptide repeats (IFITs), RIG-I-like receptors (RLRs), namely, MDA5 (IFIH1), RIG-I (DDX58), and LGP2 (DHX58), IFN-inducible guanylate binding proteins (GBP), and IFN-stimulated ubiquitin-like proteins (ISG15). Additionally, ASFV-Δ9L/Δ7R infection exhibited dramatic induction of genes important during the acute-phase response, such as IL-1A, IL-1B, IL-10, and IL-18, tumor necrosis factor alpha (TNF-α), and several members of the complement pathway (C1R). Finally, genes coding for chemokines were also more strongly induced in the ASFV-Δ9L/Δ7R-infected BMDM, including C-C motif chemokine ligand 8 (CCL8), CCL24, and C-C motif chemokine ligand 3-like 1 (CCL3L1), as well as C-X-C motif chemokine ligand 2 (CXCL2), CXCL8, and 10 CXCL10. Collectively, those data showed that combinational deletions of MGF360-9L and MGF505-7R resulted in a more pronounced innate immune response, with evidence of concurrently strong and protracted induction of genes relevant to viral recognition, IFN response, and inflammatory response.

Finally, in aiming to understand the function of DEGs, especially upregulated DEGs in ASFV-Δ9L/Δ7R- and parental ASFV-infected BMDM, the upregulated DEGs were analyzed for Gene Ontology (GO) classification and grouped into three main GO domains: Biological Process (BP), Cellular Component (CG), and Molecular Function (MF). The top 10 GO terms in each category were ranked based on the count of upregulated DEGs (Fig. 7C). The significantly enriched top 3 GO terms were plasma membrane (GO:0005886), extracellular space (GO:0005615), and extracellular region (GO:0005576), all within the GO domain of MF. Besides these, several innate immunity-related GO terms in the GO domain of MF, including innate immune response (GO:0045087), defense response to virus (GO:0051607), inflammatory response (GO:0006954), and immune response (GO:0006955), were significantly and substantially enriched. Taken together, the results show that ASFV-Δ9L/Δ7R can induce a stronger innate immune response *in vitro*.

### qPCR validated ASFV-Δ9L/Δ7R-induced innate gene expression changes.

**FIG 8:**
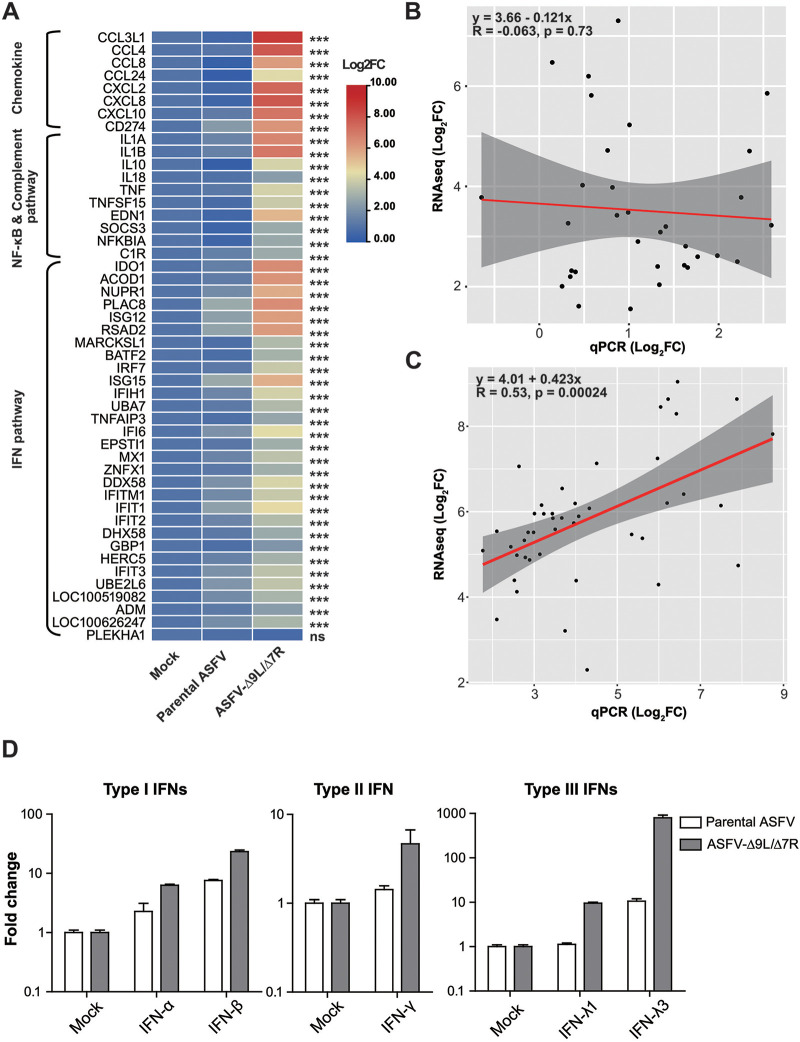
Validation of RNA-seq results. (A) BMDM were mock infected or infected with ASFV at an MOI of 0.5. A total of 48 genes encoding cytokines, NF-κB pathway, complement pathway, and IFN pathway were detected by qPCR for changes in expression with ASFV infection over time. Fold change values (relative to mock infection) were calculated using the ΔΔ*C_T_* method and are presented as the mean values in a heatmap (*n* = 3 independent experiments with replicates). Statistical analysis was performed between the results from parental ASFV-infected and ASFV-9L7R-infected BMDM. (B, C) Correlation analysis of fold changes in transcript abundance obtained from RNA-seq and qPCR analysis in parental ASFV-infected samples (B) and ASFV-Δ9L/Δ7R-infected samples (C). Gray shading denotes the 95% confidence intervals for linear regression analysis (red lines). (D) qPCR analysis of expression levels of IFNs in ASFV-infected samples over time. The results are displayed as fold change values relative to expression in mock samples (*n* = 3 independent experiments with replicates).

The transcriptomic changes detected from transcriptome sequencing (RNA-seq) during ASFV infection were further validated by quantitative PCR (qPCR). In analyzing 48 genes identified by RNA-seq as enriched in hierarchical clustering subgroups, markedly higher levels of gene expression were observed in ASFV-Δ9L/Δ7R-infected but not parental ASFV-infected cell samples, as anticipated, with the most significant changes being observed in genes encoding cytokines, with >100-fold changes in expression, and in genes encoding key components of the NF-κB pathway, displaying >10-fold increases in transcript abundance (Fig. 8A). Aiming to test the validity and accuracy of qPCR data, a correlation analysis using the log_2_(fold change) data obtained by qPCR and RNA-seq was performed. In the parental-ASFV-infected cell cultures, the qPCR data were not closely correlated with the RNA-seq data, with a coefficient of determination (*R*) of −0.063 (*P* = 0.73), mainly due to the incapability of parental ASFV to trigger substantial and significant target gene expression (Fig. 8B). Importantly, a strong correlation was recorded in ASFV-Δ9L/Δ7R-infected cell cultures, with a coefficient of determination (*R*) of 0.53 (*P* = 0.00024), further highlighting the reliability of the qPCR data in ASFV-Δ9L/Δ7R-infected cell cultures (Fig. 8C).

In the RNA-seq analysis, IFN transcripts were filtered due to low coverage in the mock-infected cell sample (fragments per kilobase per million [FPKM] < 1.0). Our qPCR analysis demonstrated that steady increases in the levels of IFN-β (type I IFN) and IFN-λ3 (type III IFN) were observed in ASFV-Δ9L/Δ7R-infected samples for an 18-h duration, consistent with the findings for other key components of the IFN pathway (Fig. 8D). Of note, type II IFN and other members of type I/III IFNs were slightly or not boosted compared to their levels in parental-ASFV-infected cell cultures. These data collectively addressed the significance of MGF deletions in triggering the host innate immune response.

### ASFV-Δ9L/Δ7R activated pathways implicated in viral recognition and antiviral innate immunity.

**FIG 9:**
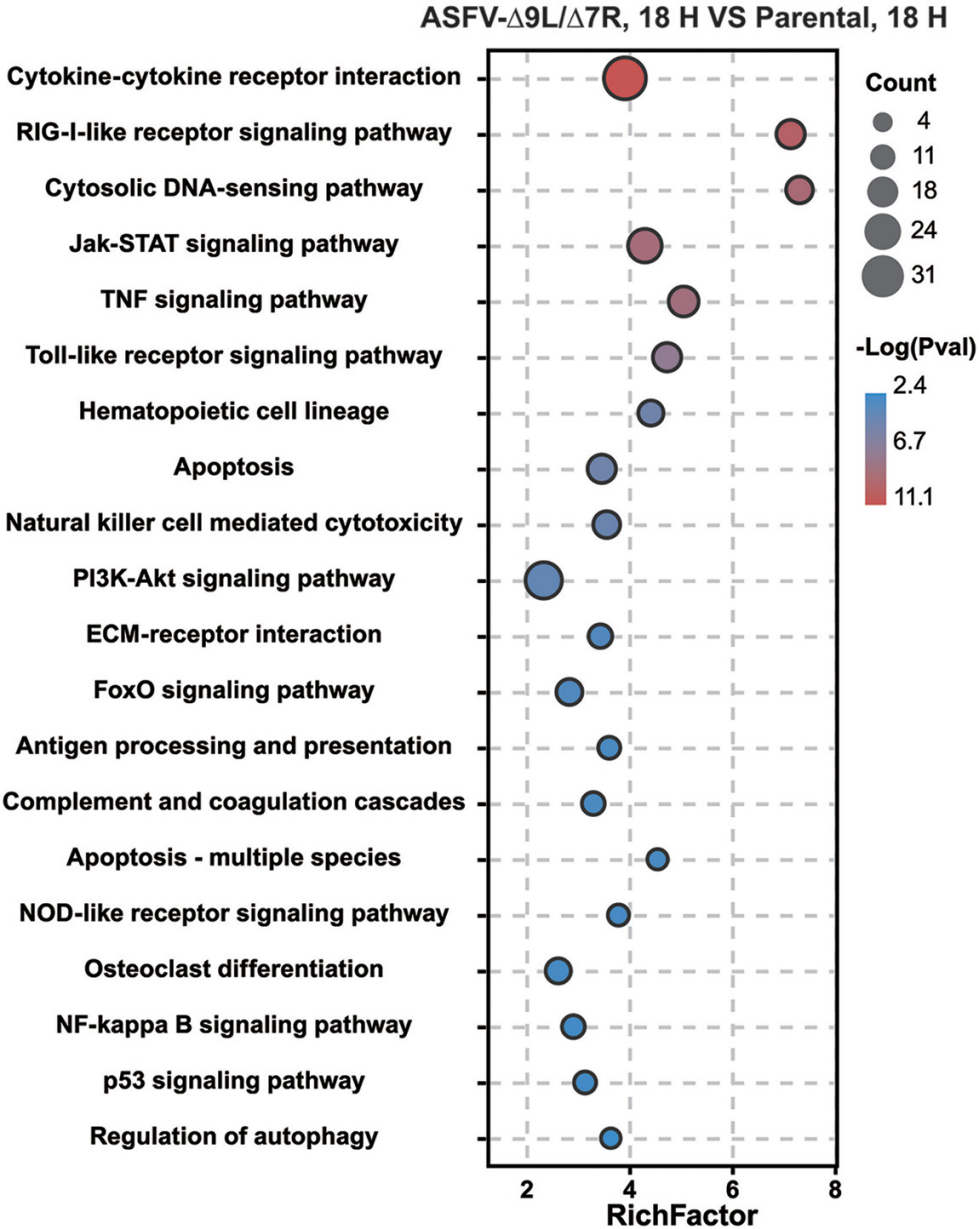
KEGG analysis.

To attempt to understand the biological pathways activated in ASFV-Δ9L/Δ7R- and parental-ASFV-infected BMDM, we mapped upregulated DEGs in the KEGG database. A subset of pathways implicated in virus recognition, including the RIG-I-like receptor signaling pathway, cytosolic DNA-sensing pathway, Toll-like receptor signaling pathway, and NOD-like receptor signaling pathway, were highly represented. Moreover, several well-known antiviral innate immunity pathways, including the JAK-STAT signaling pathway, TNF signaling pathway, apoptosis, and phosphatidylinositol 3-kinase (PI3K)-Akt signaling pathway, also displayed significant changes (Fig. 9). How the activation of those pathways constrained ASFV replication and defined ASFV pathogenesis is unknown, but these data strengthen our previous conclusion that ASFV-Δ9L/Δ7R could induce a stronger innate immune response *in vitro*.

### ASFV-Δ9L/Δ7R elicits a T-cell response characterized by CD4^+^ T cell activation and CD163^+^ macrophage infiltration in the spleen.

**FIG 10:**
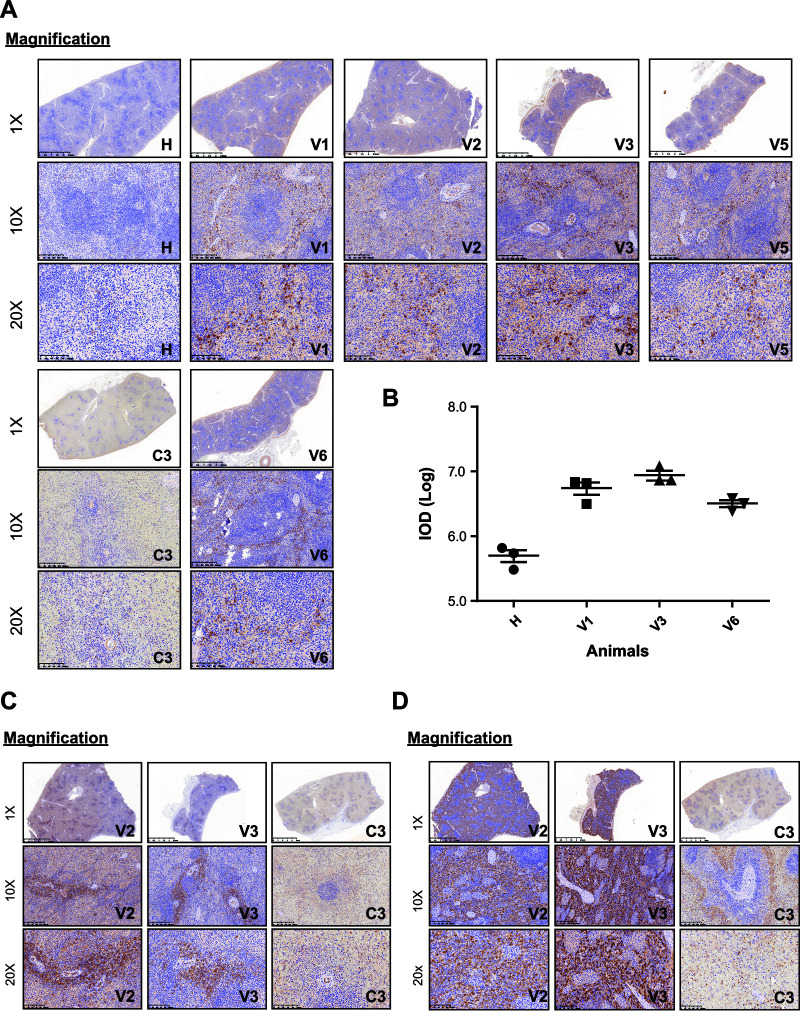
ASFV-Δ9L/Δ7R induced T cell activation and macrophage infiltration in spleens. (A) Interferon gamma (IFN-γ) status in sections of spleens from one healthy animal (H), 5 vaccinated animals (V1, V2, V3, V5, and V6), and one contact animal (C3), as shown by IHC staining. Results for IFN-γ were negative in examined sections from healthy and contact pigs. (B) Integrated optical density (IOD) results from the experiment whose results are shown in panel A. (C, D) Immunohistochemical staining of CD4^+^ T cells (C) and CD163^+^ macrophages (D) in the spleens of two vaccinated animals (V2 and V3) and one contact animal (C3). Scale bars, 100 μm.

Multiple types of data support a role for cellular immune responses in ASFV protective immunity. We next sought to evaluate ASFV-specific T-cell responses in spleens at study termination by immunohistochemistry (IHC) assays. First, strong IFN-γ immunostaining was detected in spleen samples from ASFV-infected vaccinated pigs (V1, V2, V3, V5, and V6), whereas a much weaker immunostaining signal, together with advanced autolysis, was observed in the spleen samples from ASFV-infected contact animal C3 (Fig. 10A). The integrated optical density (IOD) results paralleled the immunostaining results (Fig. 10B). Subsequently, the major porcine T-cell subsets, including CD3 (T lymphocytes), CD4 (CD-4 T lymphocytes), and CD8 (CD-8 T lymphocytes), as well as CD68 (whole macrophages) and CD163 (M2 macrophages), were immunohistochemically stained. Most of the antibodies examined failed to give the expected reactions in the spleens, probably due to low specificity. Our results primarily demonstrated that immunization with our recombinant virus elicited a T-cell response characterized by an increased frequency of CD4^+^ T cells (Fig. 10C). Additionally, high levels of CD163-positive infiltration of macrophages in response to vaccination were also detected in the spleens of vaccinated animals (Fig. 10D).

## DISCUSSION

ASFV, an emerging DNA arbovirus associated with a devastating and economically significant disease of domestic swine, has caused great losses to the pig industry globally. Until now, no commercial vaccines have been available, and strategies to combat this pathogen in commercial pig farms are desperately needed. Recent advances are showing promising results for recombinant live attenuated ASFVs with homologous or heterogenotypic cross protection. In the present study, a recombinant ASFV with combinational deletions of MGF360-9L and MGF505-7R, two well-characterized IFN inhibitors, displayed protective efficacy against homologous lethal challenge, as demonstrated by clinically normal behavior, lower levels of viremia, and better control of viral loads, as well as prevention of lesions in multiple organs. The effects of combinational deletions of MGF360-9L and MGF505-7R on improving the host innate immune response were evident, as shown by RNA-seq and qPCR assays. The boosted innate immune responses helped to initiate and shape ASFV-specific adaptive immune responses mediated by T and B cells, contributing to sterile immunity against homologous attack. Overall, our findings suggest that combinational deletions of different MGF genes with IFN-inhibitory activities exhibit a unique pattern of innate and adaptive immune responses, contributing to improved homologous protection by recombinant live attenuated ASFV.

Deletion of a single MGF gene exerts differential effects on ASFV pathogenicity. In some cases, the deletion results in an attenuated phenotype (14, 15, 28), whereas several MGF genes are not involved in virulence in swine (29, 30). MGF360-9L- and MGF505-7R-deficient mutants demonstrated reduced virulence; however, whether they could induce a protective response was uncharacterized. A previous study compared the genome sequences of nonpathogenic and pathogenic ASFV isolates from the same virus genotype and revealed that, compared to the highly pathogenic Benin 97/1 strain, the naturally attenuated OURT88/3 strain had deletions of six copies of MGF360 (MGF360-9L, -10L, -11L, -12L, -13L, and -14L) and two MGF505/530 copies (MGF505-1R and -2R) (31). The same deletions were applied in the Benin97/1 strain, along with the deletion or interruption of two more genes (MGF 505-3R and -4R), resulting in the recombinant BeninΔMGF strain. The BeninΔMGF strain could induce higher levels of IFN-β mRNA *in vitro* and induced a protective response in domestic pigs (32). In parallel, a recombinant ASFV-G-ΔMGF strain derived from the highly virulent ASFV Georgia 2007 isolate with specific and partial deletion of six genes belonging to MGF360/505 was completely attenuated and conferred protection against challenge with virulent parental virus (19). Very recently, the Chinese ASFV HLJ/18-7GD strain harboring deletions of MGF360 and MGF505 genes supplemented with CD2V has been reported to be safe and effective as a live attenuated vaccine in pigs (20). Collectively, combinational deletions of IFN modulators have proven to be a promising route for the construction of rationally designed and completely attenuated ASFV candidate vaccine strains. This conclusion is further corroborated by our findings.

We performed a detailed comparison of early host responses during infections with parental ASFV and recombinant ASFV-Δ9L/Δ7R. This study revealed boosted signaling pathways unique to ASFV-Δ9L/Δ7R that may help us decipher how these signaling molecules synergize to result in sterile immunity against lethal challenge. Of note, the type I IFNs are indispensable in eradicating invading pathogens that otherwise will be pathogenic. In the case of ASFV, *in vitro* findings demonstrated high antiviral activity of porcine type I and II IFNs, highlighting a key role in resistance to virus infection (33). Beyond the innate immune response, we also observed a close association between the presence of circulating virus-specific antibodies and protection. In our study, all vaccinated animals developed high p30-specific antibody titers upon vaccination, except for animal V4. Of note is the fact that animal V4 manifested ASFV-related clinical signs and died following challenge. However, ASFV protective humoral immune responses have remained poorly defined, especially in the context of the detailed effector mechanisms associated with and the viral proteins responsible for inducing neutralizing antibody-mediated protective responses. Exogenous injection of ASFV antibodies alone is capable of defending pigs from lethal ASFV infection (25–27). However, to date, a subset of traditional inactivated or killed ASFV vaccines have proven to be ineffective, irrespective of the inactivation method (34–36). Very recently, optimization of inactivated ASFV vaccine with state-of-the-art adjuvants failed to improve the protective efficacy in vaccinated animals (37, 38). In the case of live-attenuated ASFV viruses used as vaccines, they tended to positively affect viral immunogenicity and protective immunity, usually as demonstrated by antibody responses specific to ASFV structural proteins like p30, p54, or p72. Even though serum samples of attenuated-ASFV-vaccinated animals failed to completely neutralize ASFV *in vitro* (39), a slight inhibitory effect was observed, highlighting the elusive but solid correlation between antibody response and sterile immunity against ASFV attack.

We believe that ASFV-Δ9L/Δ7R has the potential to be developed as a strong vaccine candidate against ASFV CN/GS/2018, a circulating Chinese field strain, based on the facts of completely impaired residual virulence, apparently low potential for virus shedding and transmissibility to naive animals, and high efficacy in inducing a protective response. However, whether ASFV-Δ9L/Δ7R delivers cross-protection from challenge with viruses of the same and different genotypes needs more efforts to address.

## MATERIALS AND METHODS

### Viruses, cells, and reagents.

The ASFV CN/GS/2018 isolate of genotype II currently circulating in China was characterized and preserved by Lanzhou Veterinary Research Institute (LVRI), Chinese Academy of Agricultural Sciences (CAAS). Low-passage-number ASFV CN/GS/2018 stocks were prepared in porcine bone marrow-derived macrophages (BMDM), titrated, aliquoted, stored at −80°C, and used in all experiments described below. Porcine BMDM were prepared as outlined previously with minor modifications (40). In brief, porcine bone marrow cells were harvested from 4-week-old piglets via a series of procedures that included grinding, filtration, washing, and Ficoll density centrifugation. All cell types were cultured in RPMI Medium 1640 supplemented with 10 ng/mL recombinant porcine granulocyte-macrophage colony-stimulating factor (GM-CSF) (catalog no. 711-PG; R&D Systems, UK) to differentiate the cells toward macrophages. Following 7 days of differentiation, mature BMDM were washed, frozen in fetal bovine serum (FBS)/10% dimethyl sulfoxide (DMSO) and stored in liquid nitrogen until further use.

Immunostaining for T-cell phenotypes was performed using the following reagents: interferon gamma polyclonal antibody (catalog no. 15365-1-AP; Proteintech Group), IHCeasyCD3 ready-to-use IHC kit (epsilon chain) (catalog no. KHC0013; Proteintech Group), anti-CD3 epsilon antibody (DF6594; Affinity Biosciences), anti-CD4 monoclonal antibody (catalog no. 67786-Ig; Proteintech Group), anti-CD4 mouse monoclonal antibody (clone UMAB64) (catalog no. UM800010CF; Origene), anti-CD8a antibody (RPA-T8) (catalog no. 65144-Ig; Proteintech Group), anti-CD163 antibody (EDHu-1) (NB110-40686; Novus), anti-CD68 antibody (Affinity Biosciences; DF7518), IHCeasyCD68 ready-to-use IHC kit (catalog no. KHC0006; Proteintech Group), anti-CD163 mouse monoclonal antibody (clone OTI3B4) (catalog no. TA506386; Origene), and anti-CD163 polyclonal antibody (catalog no. 16646-1-AP; Proteintech Group). Immunostaining of ASFV p72 protein was detected by rabbit anti-ASFV p72 polyclonal antibody developed by our laboratory.

### Construction of the recombinant ASFV-Δ9L/Δ7R mutant.

Two recombinant transfer vectors were constructed in the present study. The pASFV-Δ9L recombinant transfer vector contained flanking arms (the left and right arms covered 1,500 bp upstream and downstream from MGF360-9L, respectively) and a core reporter gene cassette containing the eGFP gene under the ASFV p72 late gene promoter (p72eGFP gene cassette). Similarly, the pASFV-Δ7R recombinant transfer vector was designed using the same strategy except that the p72mCherry gene cassette replaced the MGF505-7R gene. Both vectors were engineered into the pUC57 vector. BMDM were transfected with pASFV-Δ9L using jetPEI-macrophage DNA transfection reagent (reference number 101000043; Polyplus Transfection) and subsequently infected with parental ASFV at a multiplicity of infection (MOI) of 1. At 48 h, the whole culture was gently pipetted to detach cells from the plate. eGFP-positive cells were collected under an inverted fluorescence microscope and subsequently seeded into fresh BMDM. The eGFP signals could be readily observed within 18 h and expanded further within 72 h in BMDM cells. After 10 consecutive cycles of the purification process, the single-gene-deletion mutant ASFV-Δ9L was yielded. Our double-gene-deletion mutant, ASFV-Δ9L/Δ7R, was constructed by transfecting pASFV-Δ7R into BMDM, followed by infecting the BMDM with the ASFV-Δ9L mutant. Thus, cells simultaneously positive for both eGFP and mCherry were seeded into fresh BMDM for further purification.

### Characterization of genetic modifications in the ASFV-Δ9L/Δ7R mutant.

The purity of the ASFV-Δ9L and ASFV-Δ9L/Δ7R mutants was assessed by PCR. Detection of the target genes was performed by using two primer pairs, with one flanking the target gene and the other locating within the target gene. The p72 primer pair was used as a positive indicator of the ASFV genome. Primers were designed in this study with PRIMER 3 software and are listed in Table S1.

### Next-generation sequencing of ASFV genomes.

ASFV DNA was extracted from infected BMDM using the SDS method. The harvested DNA was detected by agarose gel electrophoresis and quantified using the Qubit 2.0 fluorometer. Briefly, 1 μg of viral DNA was fragmented by sonication to a size of 350 bp, and DNA fragments were further ligated with the full-length adaptor and bar codes. The appropriate size range of the adapter-ligated library was collected and subjected to normalization of the library concentration. Sequencing libraries were generated using the NEBNext Ultra DNA library prep kit for Illumina (NEB, USA), following the manufacturer’s recommendations. The whole genome of ASFV-Δ9L/Δ7R was sequenced using the Illumina HiSeq/NovaSeq PE 150 platform at Allwegene Technology Co., Ltd. (Beijing). All good-quality paired reads were assembled using SPAdes (version 3.13.0). Finally, scaffolds with more than 500 bp were selected for subsequent analysis.

### Animal experimental design.

All animals were handled in strict accordance with good animal practice according to the Animal Ethics Procedures and Guidelines of the People’s Republic of China, and the study was approved by the Animal Ethics Committee of Lanzhou Veterinary Research Institute (LVRI), Chinese Academy of Agricultural Sciences (CAAS), Lanzhou, China. Ten 1-month-old Large White-Duroc crossbred pigs were obtained from a licensed livestock farm, with each pig being antigenically and serologically negative for ASFV. Animals were randomly assigned to two experimental groups in one pen in a room of the high-containment facilities of Lanzhou Veterinary Research Institute (LVRI), namely, a vaccination group (*n* = 6) and a contact group (*n* = 4). Pigs from the vaccination group were designated V1, V2, V3, V4, V5, and V6 and had ear tags numbered 1202, 1211, 1212, 1221, 1223, and 1224, respectively. Similarly, pigs from the contact group were designated C1, C2, C3, and C4 and had ear tags numbered 1201, 1204, 1208, and 1213, respectively. Following a 3-day acclimatization period, after physical examinations, the six pigs from the vaccination group received an intramuscular (i.m.) injection of 2 mL of cell culture medium containing 10^4^ HAD_50_ of the ASFV-Δ9L/Δ7R mutant, whereas the four pigs from the contact group were intramuscularly inoculated with the same amount of cell culture medium only. After a 23-day observational period, the four pigs from the contact group and six pigs from the vaccination group were housed separately, challenged intramuscularly with the highly virulent ASFV CN/GS/2018 strain at a lethal dose (10^2^ HAD_50_), and monitored for an extra 18 days. Throughout the entire experiment, pigs were examined daily for ASFV-related clinical signs, including rectal temperature. In addition, serum and EDTA plasma samples were collected at 2-day intervals (41), as were oral-nasal and fecal swab samples. At necropsy, tissue samples (lung, lymph nodes, heart, liver, spleen, and kidney) were examined and sampled either by snap-freezing for PCR or by fixation in 4% formalin for histology and immunohistochemistry.

### Viral titers.

Virus titers in the cell culture medium were estimated by a hemadsorption assay to quantify the endpoint dilution of the ASFV isolate on BMDM by the Reed and Muench method and expressed as 50% hemadsorption doses per mL (HAD_50_/mL) per sample. EDTA plasma and tissue samples were titrated for ASFV genome copy number using an ASFV p72 gene-based real-time PCR assay. In brief, DNA was extracted from each sample using a tissue DNA kit (D3396-02; Omega, USA). ASFV virion content was expressed as copy number by comparing the cycle threshold (*C_T_*) value of an appropriate dilution of purified DNA to sample curve *C_T_* values that were derived from a calibrated, serially diluted control viral DNA template.
