# Supplementary material for: Combinational Deletions of MGF360-9L and MGF505-7R Attenuated Highly Virulent African Swine Fever Virus and Conferred Protection against Homologous Challenge
Source: J Virol. 2022 Jul 6;96(14):e00329-22. doi: 10.1128/jvi.00329-22 (PMC9327683; doi:10.1128/jvi.00329-22)
Supplement: Supplemental file 1 — Table S1 and Fig. S1 to S5. Download jvi.00329-22-s0001.pdf, PDF file, 1.8 MB [file jvi.00329-22-s0001.pdf]

Supplementary files to

Combinational deletions of MGF360-9L and MGF505-7R attenuated highly virulent African swine fever virus and conferred protection against homologous challenge

Supplementary Table 1 List of primers and primer sequences.

Primer			Sequence
MGF360-9L	Centering primers	Forward	CATGCTGGCAATGAACGAGT
		Reverse	CGGCACAAATCTTAACGCGG
	Flanking primers	Forward	CCACTCGGTGAACAGCCTTA
		Reverse	GCTTGCCTGCGTACGATTTT
MGF505-7R	Centering primers	Forward	ACTGGCATGTTCTCCTCCCTT
		Reverse	ATTCTTTCCGCGGGATTTTCC
	Flanking primers	Forward	AGTGGAAGGCGATTTTTGGTAG
		Reverse	TGCTTGCCTAAGGGCTCAAG
P72		Forward	ATATTGCGTCTACTGGGGCG
		Reverse	AGTTCGGATGTCACAACGCT

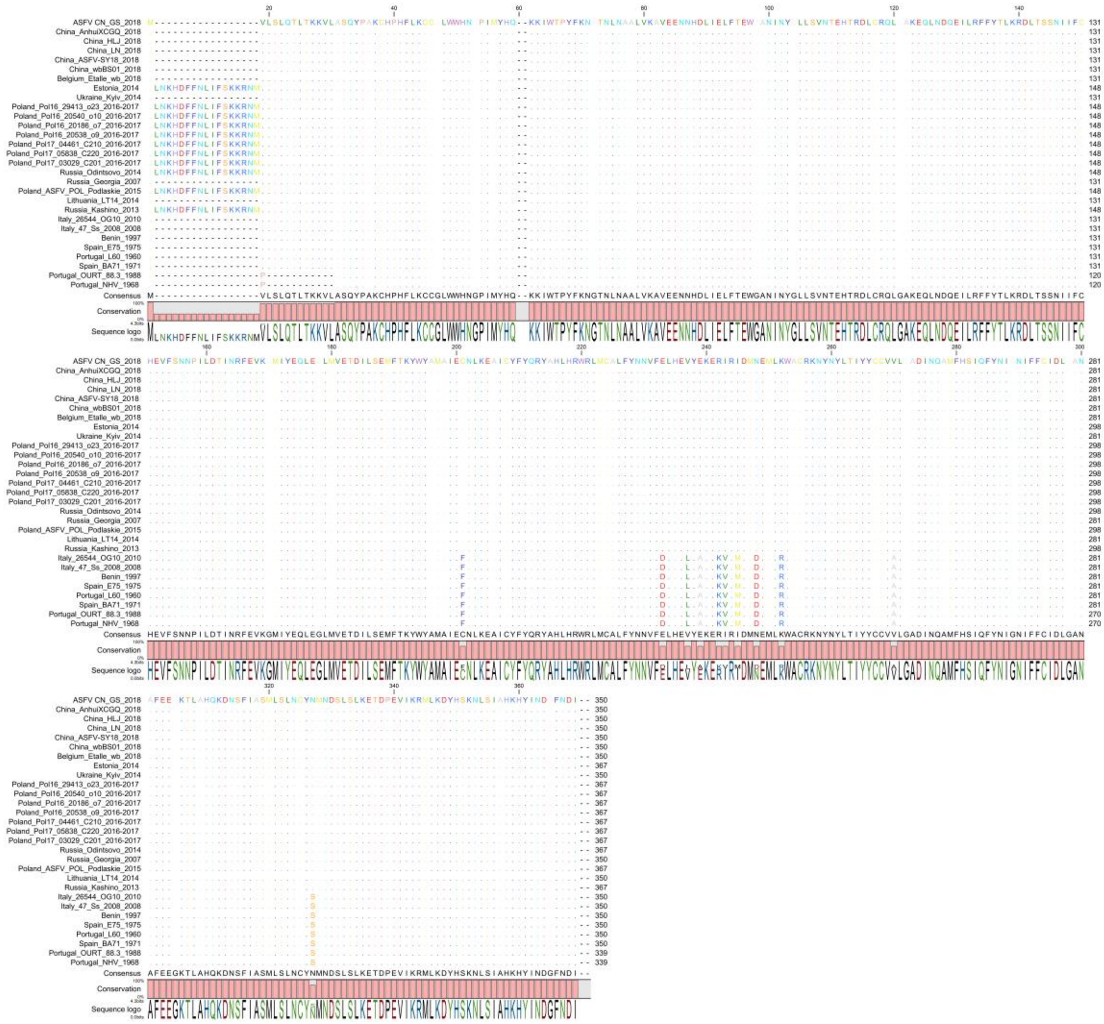

sFig. 1 Multiple sequence alignment of MGF360-9L using CLC workbench. The height of bar indicates degree of conservation across the alignment in that position with conservation scores ranging from 0% to 100%.

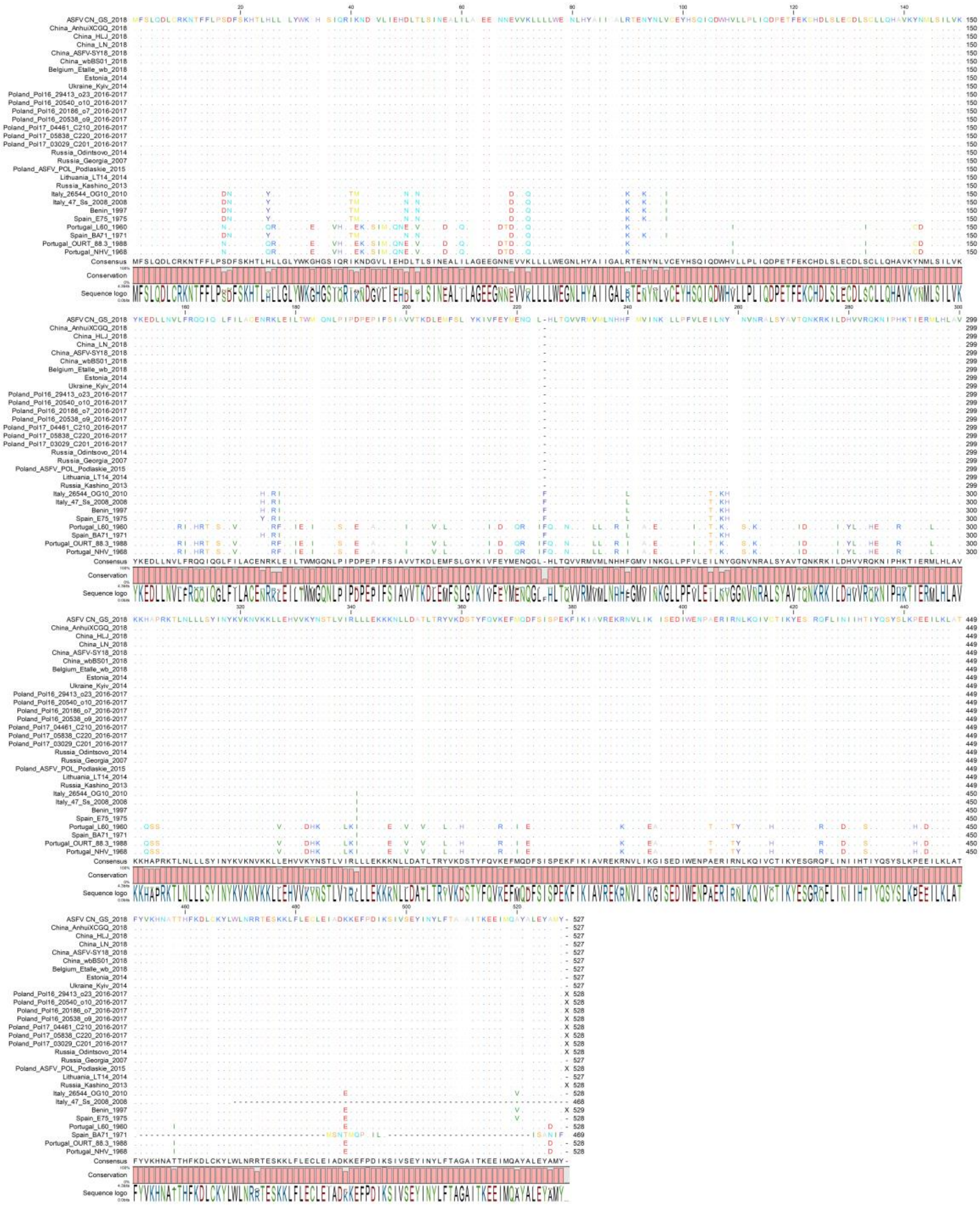

sFig. 2 Multiple sequence alignment of MGF505-7R using CLC workbench.

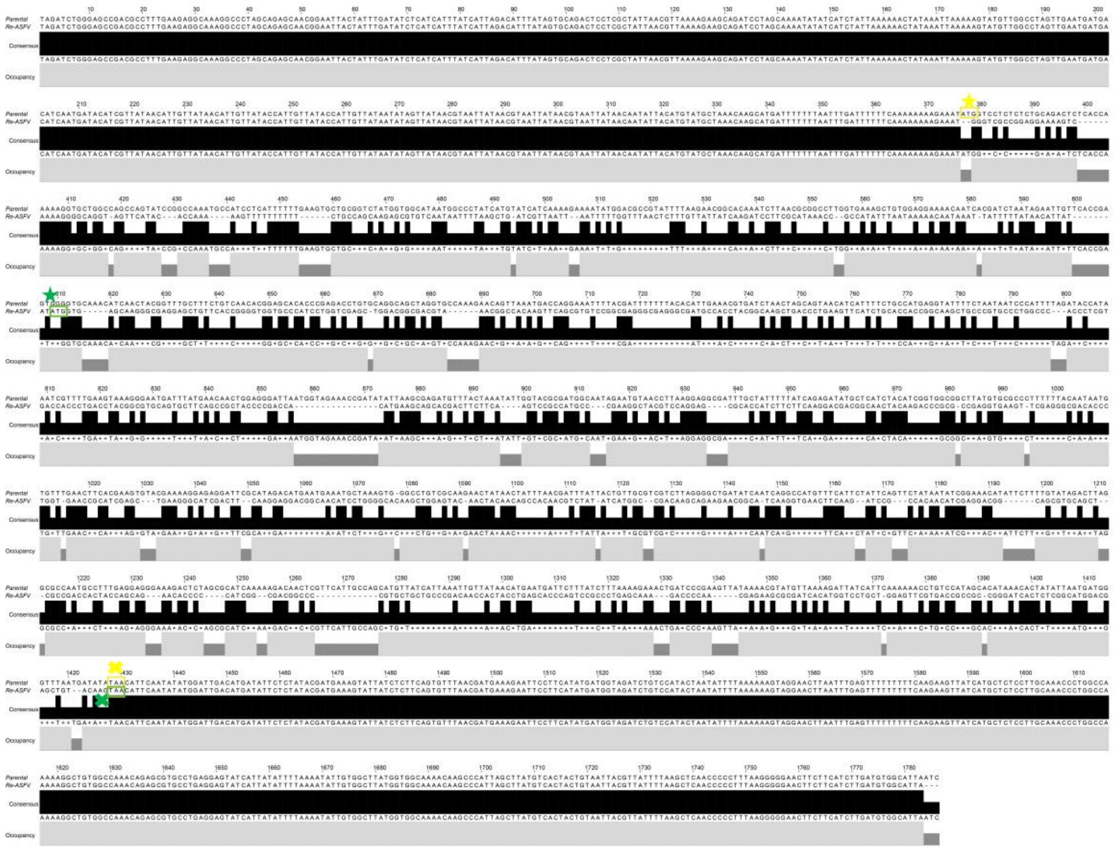

sFig. 3 Sanger sequencing validation of the precision of genetic modifications in the position of MGF360-9L gene in the ASFV-Δ9L/7R mutant. The PCR-amplified fragments with the genomic DNA from parental ASFV or ASFV-Δ9L/7R mutant were subjected to sanger sequencing. Pairwise sequence alignment demonstrated that MGF360-9L ORF was precisely replaced by p72-mGFP reporter cassette in the mutant. Yellow arrow and cross indicated the start codon and stop codon of MGF360-9L ORF in the parental ASFV, while green arrow and cross indicated the start codon and stop codon of eGFP ORF in the mutant.

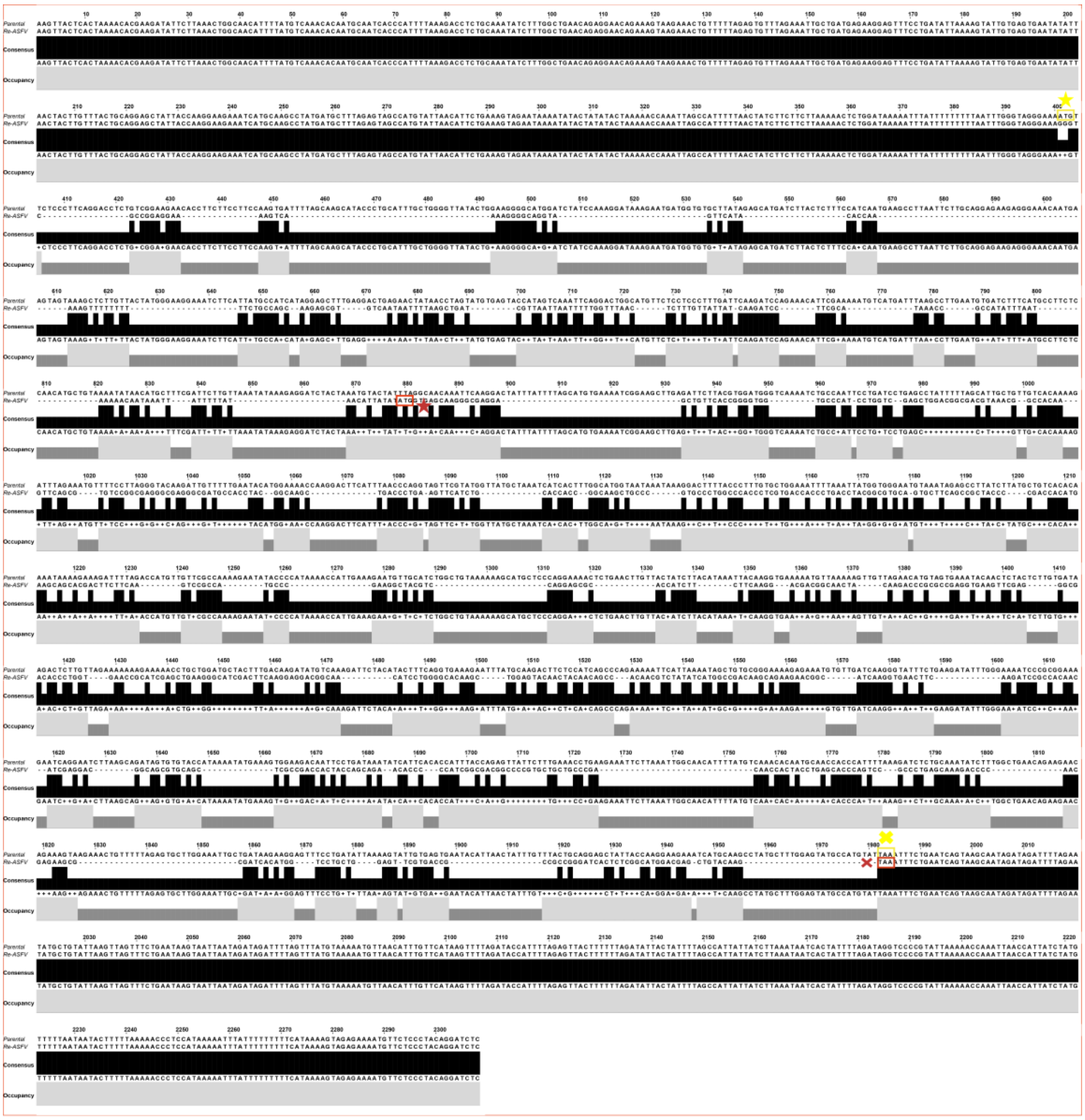

sFig. 4 Sanger sequencing validation of the precision of genetic modifications in the position of MGF505-7R gene in the ASFV-Δ9L/7R mutant. The PCR-amplified fragments with the genomic DNA from parental ASFV or ASFV-Δ9L/7R mutant were subjected to sanger sequencing. Pairwise sequence alignment demonstrated that MGF505-7R ORF was precisely replaced by p72-mCherry reporter cassette in the mutant. Yellow arrow and cross indicated the start codon and stop codon of MGF505-7R ORF in the parental ASFV, while red arrow and cross indicated the start codon and stop codon of mCherry ORF in the mutant.

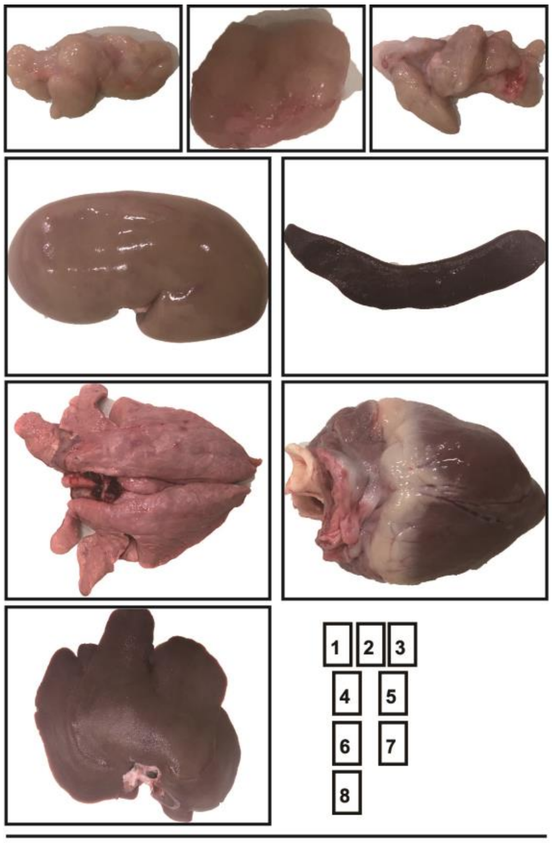

V2

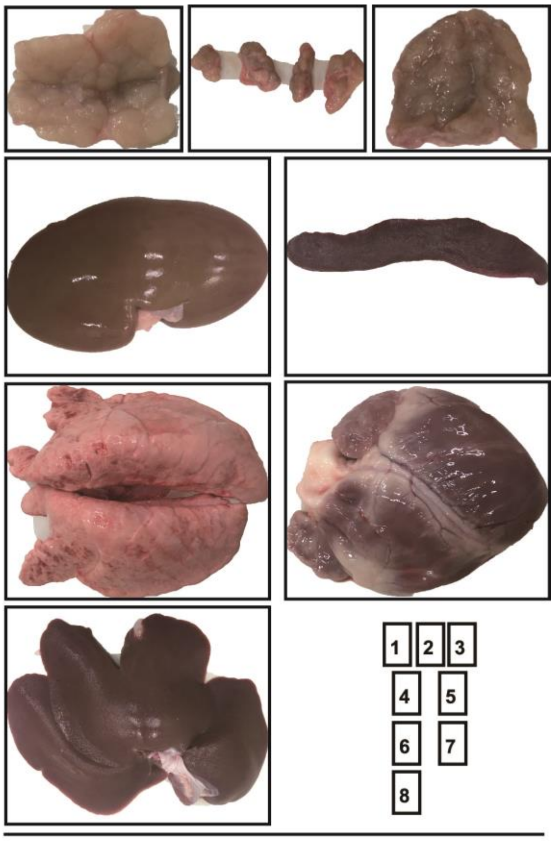

V3

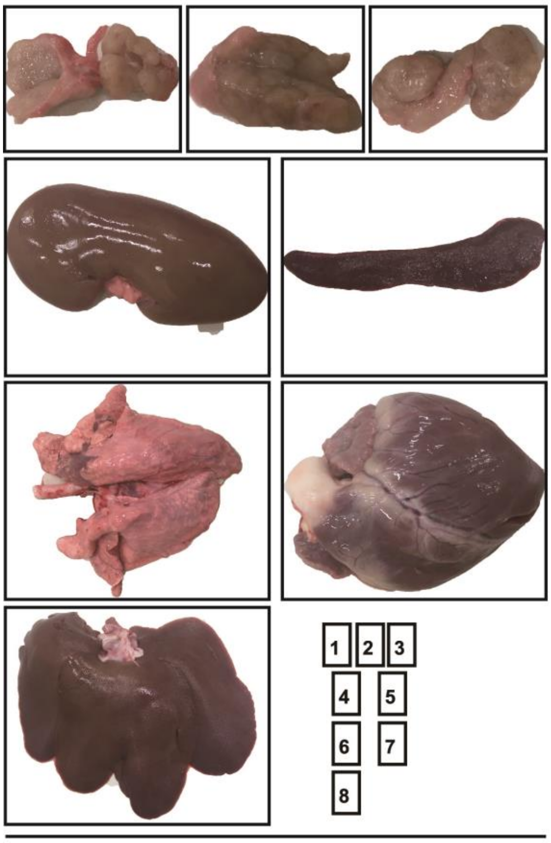

V5

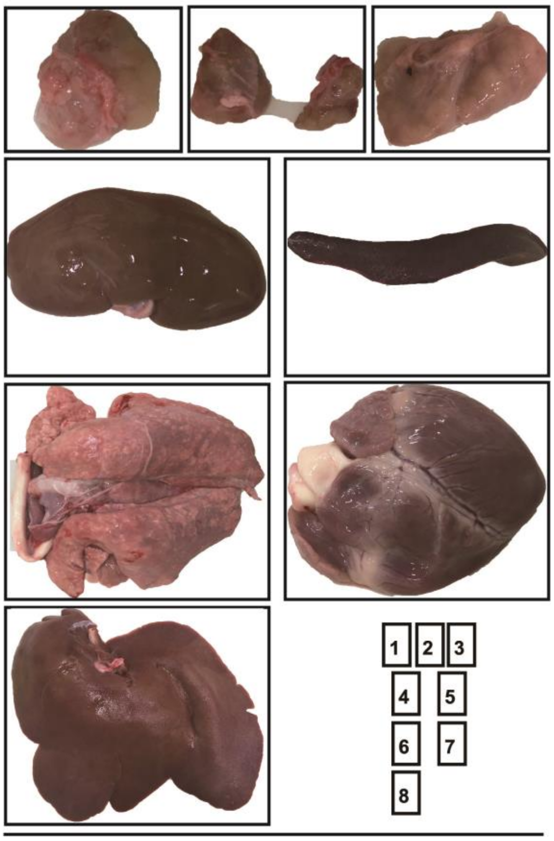

V6

sFig. 5 Postmortem lesion of ASFV on ①submandibular lymph node, ② gasto-hepatic lymph node, ③mesenteric lymph node, ④kidney, ⑤ spleen, ⑥lung, ⑦heart, and ⑧liver of vaccinated pigs (V2, V3, V5 and V6).
